# An exploration of stakeholder views and perceptions on taxing tobacco, alcohol and sugar-sweetened beverages in Ghana

**DOI:** 10.1136/bmjgh-2023-012054

**Published:** 2023-10-24

**Authors:** Arti Singh, Katherine Smith, Mark Hellowell, Divine Darlington Logo, Robert Marten, Kaung Suu Lwin, Ellis Owusu-Dabo

**Affiliations:** 1 School of Public Health, KNUST, Kumasi, Ghana; 2 School of Social Work and Social Policy, University of Strathclyde, Glasgow, UK; 3 School of Social and Political Science, University of Edinburgh, Edinburgh, UK; 4 Ghana Health Service Research and Development Division, Accra, Ghana; 5 WHO Headquarters, Alliance for Health Policy and Systems Research, Geneva, Switzerland

**Keywords:** Qualitative study, Health policy

## Abstract

**Background:**

Non-communicable diseases (NCDs) account for nearly 43% of Ghana’s all-cause mortality. Unhealthy commodities (such as alcohol, sugar and tobacco) are an important factor in the growing NCD burden in the region of sub-Saharan Africa (SSA). Despite health taxes on tobacco, alcohol and sugar-sweetened beverages (SSBs) gaining renewed attention, adoption and implementation in SSA remain limited. This study aims to unpack the contextual politics and to examine current perceptions of opportunities and barriers for health taxes in Ghana.

**Methods:**

Semistructured qualitative interviews (n=19) conducted with purposively sampled stakeholders representing four sectors: government, civil society, media and international organisations, and two group interviews with nine industry stakeholders, informed by a review of relevant literature and policy/advocacy documents.

**Results:**

Stakeholders had a general belief that such taxes are primarily useful for revenue generation (for health spending) rather than for reducing consumption and improving health. There do appear to be opportunities for health taxes with stakeholders broadly supportive of taxing SSBs. This support could be strengthened via ‘health’ framing of any new tax proposals, the generation of Ghana-specific evidence about the potential impacts of such taxes and greater public awareness. Industry actors and some government representatives opposed health taxes, citing concerns about the potential to increase illicit trade and economic harm. Some stakeholders also believed that links between politicians and affected industries represent an important barrier.

**Conclusion:**

These findings identify opportunities to introduce health taxes but also underline the potential resistance from affected industry stakeholders. Nevertheless, a strategic approach that focuses on achieving policy coherence (between central government, health and economic ministries), combined with efforts to strengthen stakeholder and public support, may weaken the lobbying position of industry. Such efforts could be supported by research to help demonstrate the value of different designs of health taxes for achieving Ghana’s health goals and to better understand industry–political links.

**WHAT IS ALREADY KNOWN ON THIS TOPIC**

- Tobacco use and alcohol and sugar-sweetened beverage (SSB) consumption are important risk factors for the development of non-communicable diseases in the African region (the latter two being more prominent challenges for Ghana).
- Existing evidence on the formulation and implementation of health taxes (targeting alcohol and SSBs) in Ghana is limited.
- Taxing consumer products that are leading risk factors could be a cost-effective way to save lives while simultaneously raising much-needed government revenue.

**WHAT THIS STUDY ADDS**

- This study represents one of the first attempts to assess key stakeholders’ positions and preferences for the use of taxes on unhealthy products in Ghana.
- Despite the presence of industry opposition and a concern about industry–political links, support for health taxes, especially taxes on SSBs, is evident.

**HOW THIS STUDY MIGHT AFFECT RESEARCH, PRACTICE AND POLICY**

- The study provides a compelling need for Ghana-specific research that explicitly links taxes to health goals to advance SSB and alcohol taxation.
- The study suggests a case for health taxes in Ghana would require close cooperation across government departments and between government, research and non-governmental organisations to successfully counter industry opposition.

## Background

Non-communicable diseases (NCDs) continue to remain the leading cause of death in sub-Saharan Africa (SSA), accounting for 37% of deaths in 2019.^1^ This includes Ghana, where NCDs account for around 43% of all-cause mortality. Premature death (between 30 years and 70 years) due to NCDs is 21% in Ghana (higher than the global average of 18%).^2^ Moreover, close to 43% of Ghanaian adults are either overweight (25.4%) or obese (17.1%), and the prevalence of overweight and obesity has been increasing since 1998.^3^ A similar trend is observed among children and adolescents (age 5–19 years), with prevalence of overweight in girls (15.1%) and boys (6.3%) and obesity in girls (3.1%) and boys (1.1%).^4^ The rising trend of NCDs is attributed to rising incomes, nutritional transition and rapid urbanisation.^5^

Tobacco use and alcohol and sugar-sweetened beverage (SSB) consumption are three leading risk factors for NCDs.^6^ Although cigarette use prevalence in Ghana is low by international standards (less than 3% of adults smoked cigarettes in 2020),^7^ Ghana remains vulnerable due to (1) tobacco industry interference; (2) increasing disposable income of people; (3) population growth and (4) a switch from cigarettes to other tobacco products, for example, shisha and electronic cigarettes.^8 9^ Alcohol consumption is also low by international standards. However, 25% of Ghanaian adults and 12.6% of adolescents^10^ indicate that they consumed alcohol in the past year, and those proportions have increased over time.^11^ For example, per capita consumption of alcohol increased between 2006 and 2019, driven in large part by an increase in beer consumption and increased lobbying by the industry.^12 13^ The consumption of SSBs in Ghana is also low by international standards but increased rapidly between 2006 and 2020.^14^ While the market volume for cigarettes (the most common tobacco product consumed) has declined from an estimated 637 million cigarette sticks sold in 2017 to 603 million cigarette sticks sold in 2022, the volumes of SSB and alcoholic beverages sold have both increased, from 446 million litres in 2017 to 542 million litres in 2022 for SSBs, and from 386 million litres in 2017 to 453 million litres in 2022 for alcoholic beverages.^15^ The food and beverage industry’s targeting of various nations in SSA, including Ghana, reinforces concerns about the country’s growing NCD burden.^16^

The World Health Assembly endorsed a package of 16 evidence-based interventions (‘best buys’) focusing on addressing major NCD risk factors (tobacco use, alcohol use, unhealthy diets and inadequate physical activity).^17 18^ One best buy is the use of taxes on products that have a negative public health impact (eg, tobacco, alcohol and SSBs) with the explicit goal of reducing consumption of such products. These taxes are considered to have the potential to reduce NCDs while advancing health equity and mobilising revenue for government budgets.^19–21^ According to WHO, increasing the prices of unhealthy commodities by 20% will decrease tobacco use by 4%–16%, alcohol by 13% and SSBs by 24%.^22^ Also, despite WHO’s recommendation of a minimum of 75% tax share of the retail price of tobacco,^23^ excise taxes on cigarette account for only about one-third of the pack price in low-income and middle-income countries (LMICs).^11^ For instance, in SSA, only Mauritius applies best practices for tobacco taxation with the highest share of excise and total taxes as a per cent of retail price of most-sold cigarette brands, in line with WHO’s recommendations.^24^ Alcohol is also subject to less stringent forms of regulation, and the alcohol industry continues to play a central role in policy making in many countries and at the global level.^25^

Despite the benefits of high taxes on these harmful commodities, most countries in SSA do not tax tobacco, alcohol or SSBs at high-enough levels to significantly discourage consumption.^26^ This is despite the existence of a large body of research on the health impact of well-designed taxes on tobacco, alcohol and sugary beverages.^27 28^ Country experiences show that tobacco excise tax increases are effective at reducing consumption in countries at all income levels.^7 29 30^ The introduction of sugar taxes in Mexico reduced sales of SSBs by 5% in the first year,^31^ and a marked increase in alcohol excise was associated with a decrease of 13% in mortality inequalities among Lithuanian men.^32^ Within the African context, increasing tobacco taxes in South Africa was associated with a decrease in consumption of about 40% between 1993 and 2003.^33^

Research analysing the economic implications of fiscal measures is vast,^34–37^ and a growing number of actors are now calling for the wider adoption of taxes on unhealthy commodities. Although the evidence base to support health taxes is dominated by high-income settings,^21^ the projected economic and health benefits of health taxes have increased calls for their use in more price-sensitive LMICs.^38^ Yet, while the economic case may be persuasive, the political feasibility of health taxes is likely to be context-specific. Examining the perspectives of relevant stakeholder groups to understand the opportunity for, and feasibility of, health tax implementation in LMICs, such as Ghana is essential.^21 36^ In light of the increasing consumption of potentially harmful commodities, the growing NCD burden and a dearth of locally generated evidence, this study aimed to examine the politics of, and stakeholder interest in, health taxes as a means of reducing NCDs in Ghana, with a view to identifying potential enablers and barriers for new health taxes.

### The current structure of excise taxes on tobacco, alcohol and SSBs in Ghana

Excise taxes are taxes that apply to a few selected commodities such as tobacco, alcohol and SSBs. Current ad valorem excise taxes (ie, calculated as a proportion of product price) are levied on cigarettes at 175% of the customs, insurance and freight (CIF) value (CIF is the value of an imported product as declared to customs on entry into a territory).^39^ However, since the CIF value is typically less than 10% of the retail price, the excise tax (the percentage of the retail price accounted for by excise) is small^7^ compared with the WHO standard for taxes.^40^ For alcoholic beverages, excise taxes vary by product between 10% and 47.5%, depending on the alcohol content.^41^ Sachet water and SSBs are not currently subject to excise tax, despite provisions for it in the 2013 Excise Stamp Act.^42^ In 2021, Ghana introduced the COVID-19 Health Recovery Levy Act, 2021 (Act 1068), which was a 1% levy on the supply of goods and services made in the country other than exempt goods or services; and import of goods and services other than exempt imports.^43^ Table 1 provides a summary of current tax structures in Ghana.

**Table 1:** Excise taxes on tobacco, alcohol, sugary beverages and water in Ghana

Product category	Tax rate as a percentage of the price CIF value (%)
Tobacco products	
Cigarettes	175
Cigars	175
Snuff and other tobacco	170.65
Alcoholic beverages	
Spirits, distilled or rectified	25
Spirits, blended or compounded	25
Spirits, for use solely in laboratories or the compounding of drugs	0
Spirits, denatured to the satisfaction of the commissioner-general	10
‘Akpeteshie’ (national spirit of Ghana, produced by distilling palm wine or sugar cane)	20
Wines, including sparkling wines	22.5
Beer, stout, other than indigenous beer, by percentage of use of local raw materials	
Less than 50% local raw materials	47.5
50%–70% local raw materials	32.5
More than 70% local raw materials	10
Malt drink, by percentage of use of local raw materials	
Less than 30% local raw materials	17.5
30%–50% local raw materials	12.5
50%–70% local raw materials	7.5
More than 70% local raw materials	2.5
Non-alcoholic beverages	
Waters, including mineral waters of all description	17.5
Distilled, bottled water	17.5
Sachet water	0

CIF, customs, insurance and freight.

## Methods

### Study design

We used qualitative semistructured interviews with stakeholders. Semistructured interviews are a suitable method for critically analysing a subject like health taxes because they allow for an in-depth examination of crucial nuances, contextual factors, values and beliefs, and individual motivations to improve our comprehension of a specific issue, topic or area of exploratory inquiry.^44 45^

### Sampling

The study frame included five sectors: government (bureaucrats within the health and finance sector), civil society organisations (CSOs) and non-governmental organisations, media representatives, international organisations, and the food and beverage industry (box 1). In total, we purposively recruited 31 stakeholders, of whom 28 agreed to participate. Of the three who did not participate, two (government officials) were unavailable for interviews and one (tobacco industry) did not respond. The concept of data saturation was used to guide the conclusion of the data collection.

Box 1 **Stakeholder organisations in the study**

Type of organisation

- Government (n=9)
- Civil society organisations (n=4).
- Media (n=2).
- International organisations (4).
- Industry (n=9).

### Data collection

All stakeholders were contacted via the WHO country office using an official email, which was subsequently followed up with a phone call to confirm participation. Interviews were conducted by AS and DDL, face-to-face (n=15) or online (n=6). Semistructured interviews were conducted with each participant in English and/or Twi between June and October 2022, with each interview lasting 40–60 min. Industry representatives insisted on group interviews (possibly due to providing a collective response) as opposed individual interviews; hence, two group interviews were conducted with industry stakeholders (n=9).

An interview guide was informed on the study objectives (online supplemental file 1), and analysis of relevant published literature and documents in the public domain (eg, policy documents and documents concerning health taxes published by interested stakeholders) was used. The preidentified themes covered in each interview were understanding and perceptions of health taxes, barriers and opportunities for introduction/expansion of health taxes, views on earmarking taxes, identifying key stakeholders for health taxes and recommendations for design of health taxes. All interviewees received written information explaining the study in advance and agreed to participate via a written consent form; most interviewees also agreed to be audio-recorded, with industry actors as the exception (we therefore took extensive notes of the group interviews with industry actors). To allow interviewees to share their views as freely as possible, we offered interviewee anonymity and did not record any personal identification.

10.1136/bmjgh-2023-012054.supp1 Supplementary data

### Data analysis

Interview recordings were professionally transcribed and checked for accuracy. An initial coding scheme was created by AS and KSm, informed by the study objectives (online supplemental file 1) and our knowledge of existing literature. Before finalising the initial coding scheme, we familiarised ourselves with the interview transcripts and created summaries of each interview, identifying patterns and generating additional codes as appropriate (online supplemental file 2). All transcripts were then coded by AS, using NVivo qualitative analysis software V.12, and a sample of 25% were crosschecked by KSu.

10.1136/bmjgh-2023-012054.supp2 Supplementary data

We applied a framework approach, a form of thematic analysis, to analyse the coded interview data.^46^ This encourages a step-by-step approach to analysis, accompanied by an effective and transparent audit trail.^47^ The final data interpretation was discussed among all authors and also sense-checked with three key informants representing CSOs and Ghana Revenue Authority (GRA).

### Patient and public involvement

Members of the public and patients were not involved in the research design, analysis and dissemination.

## Results

Our key findings are displayed to represent (1) stakeholder views (including support for/against health taxes), (2) perceived barriers and opportunities for health taxes, (3) views on earmarking and (4) recommendations for the design of any new health taxes. In each section, we present illustrative quotes that exemplify these key themes.

### Stakeholder’s views and support for/against health taxes

Stakeholders’ understanding of health taxes was limited to a belief that such taxes could be used to improve health and to generate revenue. About half of the stakeholders suggested the revenue for taxes targeting unhealthy commodities/industries should be directed towards health spending. For example, a Ministry of Health (MoH) official stated:

Health tax on the alcohol and tobacco should be geared towards improving the health of the population, and not necessary to raise revenue for government, […] a health specific tax should come to the health sector, whilst some can go to our CSOs, community level activities, to improve population health. (MoH)

Government and CSOs participants were generally supportive of health taxes and justified this on revenue generation grounds while also considering the opportunity for using the taxes to improve health (ie, depicting such instruments as win–win policies). They acknowledged that the current taxes on most unhealthy commodities (tobacco and alcohol) were not sufficiently high to affect consumption. As one stakeholder from the Ministry of Finance (MoF) argued about tobacco taxes,

Our intention was for the consumption to reduce. But that is not the case. So that is the issue that we have for now, the taxes are not deterrent [consumption] enough. (MoF)

Stakeholders representing GRA and MoH both argued on the current underfunded heath system, with most revenues directed towards the management of infectious diseases such as malaria, COVID-19 and HIV/AIDS (a situation made worse by the recent pandemic). International organisations such as WHO suggested that taxes are important for achieving Ghana’s pledge to achieve universal health coverage (UHC) by 2030. Stakeholders belonging to international organisations highlighted the same two advantages of health taxes: revenue generation and reduction in consumption of unhealthy products.

Stakeholders consistently identified the same key actors as important for understanding the political landscape surrounding debates about health taxes in Ghana. This included CSOs, the media, government (MoF and GRA), and global actors including WHO and the World Bank. Other stakeholders (from CSO and GRA) particularly highlighted the role of the Food and Drugs Authority (FDA) as key to coalescing support for health taxes in Ghana. The health ministry on the one hand was not identified as a key stakeholder for health taxes debate. Finally, there were also the respective commercial industries, which are widely recognised as active in pushing against health taxes.

The main arguments articulated against health taxes focused on the potential for negative impacts on relevant industries, including reducing employment and revenue in these sectors. Some interviewees, for example, from MoH, argued that introducing high taxes on unhealthy products might be a disincentive for industries to remain in Ghana, if their products become unaffordable to the public:

We have to realize that some of these taxes are not high enough because of the economic nature of such taxes - for employment opportunities and revenue generation, so sometimes being too hard on them may destroy people’s livelihood, which also makes people angry with the government. (MoH)

This perspective was evident in some government interviews as well as among industry stakeholders, partly due to a perception that there could be a strong pushback on any government trying to introduce health taxes (a view likely heightened when faced with global and national economic uncertainty). CSOs reflected that this was also a perspective they commonly encountered in their conversations with government representatives. For example, one CSO representative stated:

The government is not interested in this and we have been struggling to get their interest in expanding and increasing revenues for health spending, but surprisingly when you meet them, they argue in defense of the industry. (CSO)

On the other side, industry actors (alcoholic and SSBs) typically centred their arguments on the possibility for health taxes to promote illicit markets and advised the government to focus on informal sector. Their worries centred on the existence of a sizeable market for unauthorised dealers that the government fails to recognise and regulate. Additionally, they argued that current taxes on several alcoholic beverages are already high with little space for any further increment. They also stated that customers ought to have the individual liberty to choose what they want, and the current ongoing global crisis is not timely for reviewing taxes. Two industry representative said, ‘Let people make their own informed choices – it is for the consumers to decide’ (industry) and ‘Russia-Ukraine war not too good time to think about review of taxes’ (industry).

### Opportunities and barriers for health taxes

Despite concerns around government disinterest and industry opposition, the broader political landscape (some government officials, CSOs and global actors) was supportive of health taxes reflecting an opportunity. Moreover, the interview data suggest that framing new taxes in ways that emphasise their value to health could bolster support. However, stakeholders reported four main barriers to implementing health taxes; industry–political links, political and economic factors, lack of accountability, and unavailability of data and evidence (table 2).

**Table 2:** Opportunities and barriers for health taxes

Themes/subthemes	Explanation	Exemplary quotes
Opportunities for health taxes		
A supportive environment.	Almost all participants indicated that there was currently a supportive environment for health taxes based on the experience of the current debates on increasing current tobacco taxes and the involvement of strong advocacy groups (such as VALD) and the collaborative efforts of the GRA, Food and Drugs Authority and UNDP. Participants reported regular engagements/meetings between government and advocacy groups on several fronts, including tobacco and alcohol policy, and argued this level of engagement was indicative of a supportive environment. However, some CSOs and global organisations were concerned that the MoF, specifically, may not be fully supportive of this agenda. A review of current taxes on many of these commodities, including tobacco and alcohol, was recommended by global organisations such as the WHO, as the current taxes were perceived to be too low by most stakeholders. Such a review may also aid ongoing debates between different parts of the government (eg, MoH and MoF).	‘The revenue authority guys are in support, the finance ministry was a little bit less enthusiastic, maybe a little more careful, but the revenue guys you know, the revenue collectors, were aware that taxes for spirits were low and tobacco taxes were also low, and they were talking of doing something about it, getting a higher rates, but they were talking about what they want to target, if they should go for cigarettes, or how to get higher rates - so the interest is there’. (Global actor, World Bank)
Health framing (instead of revenue generation).	A health framing of any new taxes (instead of revenue generation) was generally believed to provide an important opportunity for garnering support for new taxes, especially within the context of the current COVID-19 pandemic, which made health issues more salient for many stakeholders. Global and CSO actors also attributed the rising burden of NCDs to increase in consumption of alcohol and SSBs, combined with the impact of COVID-19 on those with NCDs, thus increases salience of the opportunities presented by health taxes.	‘COVID created an opportunity for us. It pointed to us that over 90 percent of the people that died were persons with underlining NCDs and so it is a good reason to us to produce healthy products’. (CSO, revenue mobilisation)
Barriers to health taxes		
Political and economic factors.	Despite the sense among many interviewees that there was generally a positive view of health taxes among stakeholders in Ghana (as outlined previously), the industry’s (alcohol and SSBs) role in employment opportunities and their impact on the country’s economy were nonetheless a concern among some interviewees (n=8). The current economic crisis could also present a potential roadblock for any introduction of taxes in the country.	‘We have to realise that some of these taxes are not high enough because of the economic nature of such taxes - for employment opportunities and revenue generation, so sometimes being too hard on them may destroy people’s livelihood, which also makes people angry with the government’. (MoH)
Industry–political links.	The close relationship that the government is perceived to have with the industry was highlighted as an important barrier to implementing any form of health taxes. The tobacco and alcohol industries were regarded as very powerful by stakeholders and have the ability to create jobs and generate revenue. Industry was perceived by many of our interviewees to influence policy by bribing politicians and government officials. A typical example of this (focusing on the tobacco industry) was shared by CSO and journalists.	‘When we say industry, then I know people will have the picture that policymakers are different from the industry players but let me tell you some of these policy makers are in bed with some of these industry operators and some even are sponsored by these players’. (CSO, revenue mobilisation)
Lack of accountability.	About a third of stakeholders identified a perceived lack of government accountability mainly as a result of bribery and corruption. A particular instance cited was the lack of transparency in the management of the new COVID taxes.	‘I think when we had issues with COVID in the country, there was a 1% percentage points set up for COVID, but as at now, I don’t know if there is any accountability, what has been generated or what it has been used for, and all that, because that’s also an earmarked fund of which we know nothing’. (MoH)
Lack of data and evidence.	A third of the stakeholders also argued that there needed to be better local data and evidence on the role of unhealthy commodities in poor health outcomes in Ghana, in order to help make the case health introducing taxes. There was therefore an argument for more publicly accessible research on the health (and economic) impacts of alcohol and SSB consumption in Ghana.	‘We need empirical evidence to inform our bosses. Is there any data to say this number of people died as a result of tobacco or alcohol? But if we just say people are dying without any evidence, it is not helping, and this is something we hear every day’. (MoF)

CSO, civil society organisation; GRA, Ghana Revenue Authortiy; MoF, Ministry of Finance; MoH, Ministry of Health; NCD, non-communicable disease; SSB, sugar-sweetened beverage; UNDP, United Nations Development Programme.

### Views on earmarking

The MoH and CSOs generally supported earmarking taxes for health spending and, specifically, for NCD care, given that the current health insurance prioritises mainly infectious diseases. An MoH representative expressed support for earmarking as

I support it 100% if there is any such thing; it is long overdue, but in pushing for those taxes we should be specific that once these taxes are taken, they shouldn’t be put into the consolidated funds and be used for other purposes. (MoH)

As expressed by CSOs and the National Health Insurance representative, taxing unhealthy products could be an additional source of revenue especially for management of chronic conditions (not currently covered by the national health insurance). However, stakeholders from the FDA and the Ministry of Education saw a perceived lack of accountability around spending as a major challenge. Industry representatives were not in favour of earmarking either and provided the same reasons of lack of accountability of earmarked funds based on the lack of transparency as observed in the case of COVID-19 taxes.

Interviewees from international organisations, such as the World Bank and WHO, were more equivocal about the case for earmarking health taxes, arguing that earmarking could stymie the ability of governments to rapidly redirect spending to respond to emerging national needs while also acknowledging that earmarking can be useful in garnering public support for taxes. For instance, the World Bank representative said:

In budgeting it is never a good idea to earmark funds because you reduce the flexibility of the government, however, it is also true that health is chronically, underfunded in most of these countries including Ghana, and that makes me change my mind on this, an increase in taxes is acceptable to the people once it is going towards health. (World Bank)

### Recommendations for tax structure and way forward

Health taxes can be designed in lots of different ways to achieve multiple different ends, from revenue generation to the reduced consumption of unhealthy products to product reformulation.^21^ The different ways of calculating excise tax (ad valorem, proportional ethic vs specific and fixed) also have implications for the likely impacts on consumer behaviour and, potentially, for industry opposition (since different companies are likely to prefer different tax structures according to their pricing structure.^48^ Almost all the stakeholders (excluding industry) supported shifting the current tax structure away from ad valorem excise taxes (which are proportional to product price) towards more specific (fixed) taxes, or least hybrid (mixed ad valorem and specific) systems, on the basis that specific taxes result in less price variation and, therefore, less potential for ‘downtrading’ (where consumers opt for cheaper products in order to continue consuming products post tax increases).^49^ On the other hand, a GRA official notes that specific taxes are less effective when inflation is high:

In 2007, we introduced the specific tax, but you know specific taxes work well in a stable environment, where inflation is not high, because if you are not careful, specific taxes does not take into consideration the value of the products so the value of the product may go up but the taxes will remain the same. (GRA)

The revenue authority indicated that a hybrid tax system would be more effective in generating revenues as compared with a specific tax alone of which part could be earmarked for health. This variation in perspectives suggests further research may be required to understand how best to maximise stakeholder support for proposals for new/additional health taxes.

When discussing potential proposals for SSBs taxes, CSOs called for a tax system based on sugar content due to the availability of natural sugar-free juices too and were supported by others, including international organisations:

Well you know, when we are proposing, for SSBs taxes, we also have juices that do not contain sugar – the natural ones, there should be some arrangements for that, because it is the sugar that we are talking about here. (CSO)

Industry stakeholders generally did not comment on tax structures or rates, opposing the idea of health taxes wholly (though this may simply reflect the fact we interviewed different companies, which might well have difference preferences, collectively).

Recommendations for alternative (or additional) strategies to prevent the consumption of unhealthy products are presented in table 3.

**Table 3:** Recommendations for preventing consumption of unhealthy products

Recommendations	Exemplary quotes
Need for more data and evidence including stakeholder engagement To generate evidence including public opinions on harmful products and taxing on alcohol and SSBs and present findings to policy makers to influence change	‘I will recommend that we get research in these areas, we already have research works in tobacco which is excellent, do we have studies in the area of beverages, and alcoholic areas, no, maybe we have done the research alright, but have we presented it to policy’. (GRA)
Reduce industry and political connections Reduce the interactions at all points and prevent any influence of the industry on policy.	‘I think the argument that they are creating jobs and doing social responsibilities, we should pin them down by saying yes you have created jobs but you have also created a big mess to the citizenry, and the mess should drop you from a strong work force and from making more profits, but they will always come with arguments, and we need to counter argue and ensure that there should not be any interactions with government when it comes to influencing policy’. (CSO)
Invest in public education on and awareness of unhealthy commodities This was recommended as a key strategy with incorporation of religious and community leaders.	‘Some community advocacy programs must be initiated, we have to get some chiefs involved, we know how powerful chiefs are, we also know how powerful the religious community are, the Christians and the Muslims’. (Media)
Reducing the advertisements of harmful products such as SSBs in the media	‘Apart from the tax measures there are other measures such as the ban on advertisements, such as advertise after 8pm to make sure that certain population groups especially the youth, the kids are not exposed to these kind of things so that they don’t get involved at an earlier stage’. (GRA)
Reformulation of sugary beverages to lower the content of sugar	‘Coke which uses a lot of sugar, they reacted with coke zero, and now they are saying that coke has no sugar. Can we push our local manufacturers to come up with their product zeros, where they can say that we are now moving towards health, where health means that their products are without sugar’. (Customs)
Marketing strategy This is based on not taxing healthy foods to increase their uptake and reduce consumption of unhealthy products.	*‘*In licensing these food outlets such as KFC or Eddys Pizza and others, we should put the health tax component in there in licensing or renewing their license to operate and that could be earmarked for health and it should be graded, for instance if you go to a place that sells natural fruit juice, the health tax should have a base line, and once you go to an outlet where they sell these alcoholic or sweeten beverages, then probably a percentage should be added, and it must be discriminatory in nature’. (MoH)

CSO, civil society organisation; GRA, Ghana Revenue Authortiy; MoH, Ministry of Health; SSB, sugar-sweetened beverage.

## Discussion

This qualitative study brings together narratives of actors representing the health and finance ministry, CSOs, international organisations, media and industry, on the contextual politics surrounding health taxes in Ghana. Findings indicate a general belief that such taxes are primarily useful for revenue generation (for health spending) rather than for reducing consumption and improving health. With the exception of industry actors, government, CSOs and media representatives supported the need for taxes to be high enough to affect consumption patterns. Some stakeholders (such as the health ministry) also cautioned that governments are hesitant towards health taxes as these would harm the economy’s ability to thrive and would negatively impact on important industries. Likewise, industry actors largely opposed health taxes, pointing to concerns about the promotion of illicit trade and other economic harms. Nevertheless, there do appear to be opportunities for health taxes in Ghana, with stakeholders broadly supportive of taxing SSBs and tobacco (and alcohol, to a lesser extent). While these views were expected (reflecting existing literature), concerns about the links between politicians and affected industries were observed.

Close to two decades since the WHO Framework Convention on Tobacco Control was enacted,^50^ there is convincing evidence that the single, most effective approach to decrease tobacco use and save lives is to raise tobacco prices through taxation.^29^ However, evidence on alcohol and SSBs is still emerging,^26^ and there remains little research on why and how LMICs, particularly in SSA, use fiscal measures. As the primary focus of taxes on unhealthy commodities (such as alcohol and tobacco) in African nations such as Ghana are for the fiscal revenues they generate,^16^ taxes on alcohol and tobacco products have been set at rates comparable to other consumer products, rather than at rates designed explicitly to reduce consumption.^51^ The benefit of introducing fiscal measures on these unhealthy commodities is extensive^19 52 53^ and can be a starting point for many African countries, including Ghana, where there is a lack of country-specific evidence.

While stakeholders in our study were generally supportive of health taxes through the presence of an enabling environment (ongoing stakeholder discussions for tobacco tax review) and global or regional momentum (by the Economic Community of West African States and the United Nations Development Programme), a strong industry presence (mainly alcohol and SSBs) was observed. Similar to countries such as Uganda^54^ and Rwanda,^55^ perceived tensions exist between health and economic policies. Nevertheless, despite industry efforts and existing tension within ministries, taxation is gaining more attention from policy makers as a win–win–win policy measure for public health, domestic resource mobilisation and equity (although contested).^36^

The key actors operating in the domestic tax policy implementation ecosystem in the country were mainly the MoF, GRA and CSOs, including VALD (have had an active role in tobacco control for many years), NCD Alliance and Alcohol Policy. The role of CSOs in taxes in Ghana has been well documented,^56–59^ a role that all of our key informants acknowledged. VALD and Ghana NCD Alliance are at the forefront of advocacy on taxing unhealthy goods (especially tobacco).^60–62^ Recently, the Ghana NCD Alliance, in close cooperation with VALD, promoted the mainstreaming of NCD prevention and control in the national development framework in line with the Sustainable Development Goals.^63^ Through their advocacy, the MoH has included financing health by taxing health harming products in the 2022 National NCD Policy and Strategy plan as well as a recommendation to establish NCD funds.^64^ It is expected that the recognition of tax increment on tobacco, alcohol and other unhealthy products will empower the MoF to advance the call for a legislation on tax increment to finance health and development priorities. Literature examining CSO perspectives on health taxes in SSA is limited, even though their engagement has been documented.^65^ Contrary to the scenario in Ghana, Sharp *et al*’s study on CSOs in Kenya, Nigeria, Uganda and Zambia indicated that CSOs may oppose taxes and that business associations tend to be more active in lobbying around tax reforms than CSOs.^65^ For instance, in Uganda, the government remains hostile to CSOs, despite some government officials facilitating CSO activities.^65^ This is an important consideration for any attempts to introduce new or increased health taxes in SSA since it suggests that affected industries will push back strongly, while support from CSOs cannot be assumed to be universally favourable by governments.

It was not surprising that there was support for SSB taxes from almost all stakeholder groups except industry actors. This has been the case with many nations internationally including SSA that have considered an SSB tax and has had to contend with the influence of powerful multinational corporations (such as Coca-Cola and PepsiCo), which have an increasing presence in African nations as growing markets.^16^ One of the major drawbacks in many countries in SSA is the lack of local data (as indicated by many stakeholders), and countries in SSA are not in a strong position to use local data to support the case for SSB taxes.^38^ The availability of good data is critical for influencing policy and decision making as shown in countries like Mexico^34^ and the UK.^35^ Ghana currently has an SSB tax on water and other beverages with or without sugar.^66^ It may seem perverse to have an excise tax on bottled water but not SSBs. Given rising obesity and other NCDs in Ghana, an SSB tax might be an effective fiscal policy to decrease purchase and consumption of SSB and reduce overweight/obesity prevalence, especially if the tax were specific for beverage volume.^67^ Several strategies that have been identified to support the implementation of SSB taxes include shifting the discourse from an economic to a health perspective, developing positive public opinion, garnering public support, forging links with the agricultural sector and central government leadership including MoF.^68–71^

Earmarking is a tool of public health policy that charges the consumption of unhealthy products like alcohol and tobacco. In general, health policy makers are likely to support earmarking, such taxes for health spending, while officials within finance ministries are likely to oppose earmarking commitments.^37 68^ In our study, respondents belonging to both health and finance ministries and CSOs were supportive of earmarking funds for health spending on NCDs. However, it was argued that earmarking may not result in a significant and sustained increase in the priority placed on health in overall government spending (mainly due to poor accountability of taxes in general). As budgets are fungible, allocating one revenue source is likely to result in offsets from other sources. For instance, after over 15 years of earmarking to fund Ghana’s National Health Insurance, concerns are emerging that other health priorities, such as immunisation programmes, may be suffering.^21^ including challenges related to expenditure management and ensuring earmarked funds bring value for money in the NHIS.^72^ Earmarking can also enable policy makers to respond to public concern with regard to the regressive nature of consumption taxes, via commitments to target revenue spending at the poorest social groups.^21^ A report by the World Bank found that the large financing gap for UHC in LMICs (now exacerbated by the economic effects of the pandemic and economic crisis) could be mitigated by tax increases on tobacco, alcohol and SSBs.^73^ Prior to the COVID-19 pandemic, the Ghana government was initiating reforms aimed at achieving UHC but identified financing as a persistent challenge.^74^ In light of this, there is the need for a more thorough exploration of earmarking taxes for specific health spending for chronic diseases and exploration of the operational considerations behind how earmarks are managed and operated in the country. One key strength of the study is its timing. The study coincides with ongoing discussions in Ghana about revising excise taxes on tobacco, alcohol and SSBs.^75–77^ In response to the worsening economy of Ghana, the government introduced three new taxes in March 2023—the Excise Duty Amendment Bill 2022, Growth and Sustainability Levy Bill and the Income Tax Amendment Bill 2022—to raise revenue and meet the criteria for a $3 billion International Monetary Fund programme staff-level agreement. The objective of the Excise Duty (Amendment) Act 2023 (Act 1093) (table 4) is to revise the excise duty for tobacco products, wine, malt drinks and spirits and to impose an excise duty on sweetened beverages (including fruit juices), electronic cigarettes and electronic liquids.

**Table 4:** Excise duty Amendment Bill 2022

Product category	Tax rate as a percentage of the price CIF value
Tobacco products	
Cigarettes	50% of the ex-factory price
Cigars	50% of the ex-factory price
Snuff and other tobacco	Ghana Cedis 280/kg
Electronic cigarettes	50% of the ex-factory price
Electronic smoking devices	50% of the ex-factory price
Alcoholic beverages	
Spirits, distilled or rectified	50% of the ex-factory price
Spirits, blended or compounded	50 %
Spirits, for use solely in laboratories or the compounding of drugs	0%
Spirits, denatured to the satisfaction of the commissioner-general	10% of the ex-factory price
Akpeteshie (national spirit of Ghana, produced by distilling palm wine or sugar cane)	20% of the ex-factory price
Beer, stout, other than indigenous beer, by percentage of use of local raw materials	
Less than 50% local raw materials	47.5% of the ex-factory price
50%–70% local raw materials	32.5% of the ex-factory price
More than 70% local raw materials	10% of the ex-factory price
Cider beer	20 per centum of the ex-factory price
Wines including sparkling wines	45% of the ex-factory price
Malt drink, by percentage of use of local raw materials	
Less than 50% local raw materials	20% of the ex-factory price
50%–70% local raw materials	12.5% of the ex-factory price
Above 70% local raw materials	10% of the ex-factory price
Non-alcoholic beverages	
Waters, including mineral waters of all description	20% of the ex-factory price
Distilled, bottled water	17.5% of the ex-factory price
Energy drinks	20 per centum of the ex-factory price
Fruit juices (including grape and vegetable juices, unfermented and not containing added spirits whether or not containing added sugar or other sweetening)	20 per centum of the ex-factory price
Other non-alcoholic drinks	20 per centum of the ex-factory price

CIF, customs, insurance and freight.

This study therefore directly links the research to real-time social, political processes and stakeholder engagements associated with tax implementation. Our study also reflects a wide range of subjects, covering a sample of relevant stakeholders including the industry, who may have any kind of interest connected with health taxes. Nonetheless, there is no guarantee that opinions expressed by the interviewees are fully consistent with the views of all the representatives of given communities or organisations. Second, our positions undoubtedly influenced whom we were able to interview, especially since we could only conduct interviews in English and Twi, and how we were positioned to interpret data from contexts other than our own. To address this limitation, we incorporated some sense-checking of our emerging analysis with three key stakeholders (see the Methods section).

## Conclusion

Increased taxes on tobacco, alcohol and SSBs could achieve both vital health gains and significant revenue for Ghana, but they must be designed for maximum effectiveness and must also garner sufficient political, policy and public support to be feasible. Our findings indicate that the current Ghanaian policy environment is broadly supportive of using health taxes for NCD prevention, but, as seen in many other nations such as South Africa and Uganda, there is likely to be strong political and industry opposition. The example of South Africa and Morocco could serve as important learning lessons, where it took the combined efforts, resources and courage of civil society, academia and government to overcome food companies’ resistance to SSB taxes in 2019.^28 78^ Nevertheless, the most important next actions identified by stakeholders were to focus on generating broad support for health taxes by increasing public education and awareness on the harms caused by these products and how taxation can reduce consumption in addition to gathering more local data on the negative effects of SSB and alcohol use.

A greater emphasis on empirical research that seeks to understand the context-specific power dynamics and political complexities associated with the design and implementation of fiscal measures in Ghana and SSA seems essential. The creation of a compelling case for SSB taxation as a means of reducing NCDs requires better national data (eg, up-to-date evidence of the health and economic impacts of SSBs, projections of the likely impact of different tax designs for revenue and health, and research examining public support to complement this study of stakeholder views). Further, a supportive coalition is required, and this, in turn, requires a more thorough exploration of various design options (as is being done for tobacco) for alcohol and SSB taxes in Ghana. All of this needs to be examined in the context of the current political landscape governance and also the recently passed Excise Duty (Amendment) Bill 2022.

## Data Availability

Data are available upon reasonable request. Data will be made available upon request from artisingh_uk@yahoo.com.
