# Supplementary material for: An exploration of stakeholder views and perceptions on taxing tobacco, alcohol and sugar-sweetened beverages in Ghana
Source: BMJ Glob Health. 2023 Oct 24;8(Suppl 8):e012054. doi: 10.1136/bmjgh-2023-012054 (PMC10255295; doi:10.1136/bmjgh-2023-012054)
Supplement: Supplementary data [file bmjgh-2023-012054supp001.pdf]

Supplementary file 1: Interview Guide for Health Policy Analysis for Health taxes – Lessons from Ghana

Good morning/afternoon Mr/Mrs (Surname). Thank you for taking the time to meet with me today, I really appreciate it. I would like to start by formally introducing myself and briefly explaining the purpose of my research project and what you can expect from today's interview.

My name is ______________________. Our team is conducting a research in collaboration with the WHO; to gain a better understanding of the perceptions of various stakeholders in health taxes on tobacco, alcohol and sugar sweetened beverages in Ghana in order to provide new evidence to review the current taxes and identify challenges and opportunities associated with new health tax proposals to improve overall health of the population Ghana. It is important to understand stakeholder's perception and recommendations. This information is necessary for implementing future policies of the commercial determinants of health and for assisting the Ghana government to combat over nutrition and under nutrition during the current economic climate.

The reason I am interviewing you today is because you are a subject matter expert within your sector, and I believe that I can learn a lot from both your knowledge and experience.

Today's interview will not take more than 45-60 minutes of your time. I would prefer if today's interview could be very open, conversational and informed. As you are the professional, I would like to learn as much as possible from you. The interview is, and will remain, completely confidential and anonymous – your name and company will not be identified in the research report. As such I will not be referring to you as by your real name during the interview.

I would like to now ask for your permission to audio-record our conversation; I will be using both a recorder/phone as recording devices. Before we begin, I would like to ask if you could please read through and sign the informed consent form, including the section on consenting to being audio-recorded.

Do you have any questions with regards to today's interview before I proceed?

[Terminology: 1 We define health tax proposals as proposals to introduce or increases existing taxes on health-damaging products in pursuit of explicit population health objectives.

For the purpose of today's interview, I will be shortening sugar-sweetened beverages to SSBs.]

1. Demographics (hard copy for the participant)

1.1 Gender – Male / Female (Circle and say gender in audio-recording)

1.2 What is your date of birth?

1.3 What is your highest level of education?

1.4 What is your general job title?

1.5 How many years of experience do you have in your relevant field / sector?

1.6 Could you please talk me through what your role is in your sector?

1. There is a lot of discussion about health taxes but people sometimes mean different things – what do you think makes a particular tax a health tax?

• Can you briefly tell me about what you know on the current taxes on tobacco, alcohol and SSBs?

• How would you describe the current position on taxing alcohol, tobacco and SSBs in Ghana?

• Do you think it's more important that a health tax achieves improvements in population health or provides revenue for government spending?

• How much government interest would you say there currently is in developing/increasing health taxes in Ghana.

o Probe: Does this interest vary by Government Department?

2. Who would you say are the key stakeholders for health taxes in Ghana and how would you summarize their views?'

• How supportive of new/higher health taxes do you feel members of the public currently are?

o Probe: How open to change do you think public views are for this issue?

• How supportive do you think key media outlets are of health taxes?

• Who are the key industry actors with an interest in health taxes in Ghana?

o Probe: How influential do you think these actors are on the issue of health taxes?

• Who are the key CSO/NGO actors with an interest in health taxes in Ghana?

o Probe: How influential do you think these actors are on the issue of health taxes?

• What about the interest of global actors (e.g. World Bank, WHO but also major donors) towards health taxes in Ghana?

o Probe: How influential are these actors?

3. To what extent would you support the introduction and/or expansion of health taxes in pursuit of population health objectives?

Prompts:

- What would you say are the advantages and disadvantages of health taxes from a government revenue perspective?
  - o Probe: In general?
  - o Probe: Specifically, in the COVID/post-COVID context in which public expenditure is set to fall sharply?
- What are the benefits of health taxes in achieving key health related outcomes?
- Are there any other countries that have experimented with health taxes that you are aware of?
  - o Probe: Do you think there may be useful lessons for Ghana to draw on from those countries?
- To what extent do you feel there is a strong evidence-base for developing/expanding health taxes in Ghana?
  - o Are there any limitations of the current evidences that have?

4. What are the key barriers or challenges for introducing new/additional health taxes in the context of national political and societal dynamics?

- Political
- Administrative (e.g. would the MoF/MoH support this? Why?)Why not?
- Social
- Cultural
- Economic
- To what extent do you feel it is possible to overcome these challenges and what would need to happen to enable this?

5. What would you say the biggest opportunities for new health taxes in the context of national political, administrative and societal dynamics currently are in Ghana?

- Think of this in terms of earmarking them for National Health Insurance and/or government health services in the pursuit of health system goals, such as Universal Health Care
- Reduced demand for health-damaging goods
- Sustainable financing for public health services (e.g. in context of COVID?)
- Improvements to the physical environment
- Socio-economic status of population

6. How feasible would you say it is that Ghana develop and implement SSB taxes? (Ask CSO???)

- Administrative capacities and barriers
    - Probe: Does state capacity exist to implement such taxes effectively; if so, where;
    - If not, how could it be built?
- To what extent might the legal framework present a challenge?
- Would it be feasible to generate sufficient public support?
- To what extent do you feel it would be feasible to manage industry interference/opposition?

7. What are your recommendations for the design of a new health tax in the context of Ghana?

- Would it focus on SSB or something else?
- Which kind of tax regime do you feel would be most likely to generate political, public, administrative, industry support? (ad valorem/specific/mixed or any other)

8. Do you have any alternative measures to reduce the rising non-communicable disease burden (obesity/diabetes/cardiovascular disease etc.) in Ghana?

Probe: Or any other ways to increase public funds that may help to support other health policy objectives?
