# Supplementary material for: An exploration of stakeholder views and perceptions on taxing tobacco, alcohol and sugar-sweetened beverages in Ghana
Source: BMJ Glob Health. 2023 Oct 24;8(Suppl 8):e012054. doi: 10.1136/bmjgh-2023-012054 (PMC10255295; doi:10.1136/bmjgh-2023-012054)
Supplement: Supplementary data [file bmjgh-2023-012054supp002.pdf]

BMJ Publishing Group Limited (BMJ) disclaims all liability and responsibility arising from any reliance placed on this supplemental material which has been supplied by the author(s)

Supplementary file 2: Summary of initial emerging themes and sub-themes from the data

Themes	Sub-themes	Categories
1. Support for introduction/expansion of health taxes	1.1 Arguments in favor	1.1.1 Contribution to a healthier environment
		1.1.2 Government interest
		1.1.3 Revenue generation
		1.1.4 Consumption of unhealthy commodity
		1.1.5 NCDs and COVID-related burden
	1.2 Arguments against health taxes	1.2.1 Preferences of the consumer
		1.2.2 Impact on industries and jobs
		1.2.3 Effect on revenue generation and consumption of commodity
		1.2.4 External factors (eg. illicit trade and Russia-Ukraine war)
	1.3 knowledge and understanding of health taxes	1.3.1 Knowledge of health taxes
		1.3.2 Current tax landscape
		1.3.3 Role of key actors
	1.4 Perceived Public views	1.4.1 Socio-cultural factors
		1.4.2 Public support
2. Barriers and opportunities for health taxes	2.1 Barriers	2.1.1 Industry push back
		2.1.2 Lack of accountability
		2.1.3 Unavailability of data and evidence
		2.1.4 Political and economic factors
	2.2 Opportunities	2.2.1 Health framing (rather than revenue)
		2.2.2 Supportive environment
3.Views on design of health taxes	3.1 Type of commodity	3.1.1 SSB taxes
		3.1.2 Focus on tobacco/alcohol
	3.2 Type of tax design	3.2.1 Specific
		3.2.2 Ad valorem
4. Way forward	3.1 Recommendations for health taxes	
	3.2 Earmarking health taxes	
	3.3 Alternative measures for NCDs	
	3.4	

Singh A, *et al. BMJ Global Health* 2023; 8:e012054. doi: 10.1136/bmjgh-2023-012054
